# Enumeration of CD4^+^ T-Cells Using a Portable Microchip Count Platform in Tanzanian HIV-Infected Patients

**DOI:** 10.1371/journal.pone.0021409

**Published:** 2011-07-06

**Authors:** SangJun Moon, Umut Atakan Gurkan, Jeffrey Blander, Wafaie W. Fawzi, Said Aboud, Ferdinand Mugusi, Daniel R. Kuritzkes, Utkan Demirci

**Affiliations:** 1 Demirci Bio-Acoustic MEMS Laboratory, Center for Biomedical Engineering, Brigham and Women's Hospital, Harvard Medical School, Boston, Massachusetts, United States of America; 2 Department of Epidemiology, Harvard Medical School, Boston, Massachusetts, United States of America; 3 Department of Nutrition, Department of Epidemiology, Department of Global Health and Population, Harvard School of Public Health, Boston, Massachusetts, United States of America; 4 Department of Microbiology and Immunology, Muhimbili University of Health and Allied Sciences, Dar es Salaam, Tanzania; 5 Department of Internal Medicine, Muhimbili University of Health and Allied Sciences, Dar es Salaam, Tanzania; 6 Section of Retroviral Therapeutics, Brigham and Women's Hospital, Harvard Medical School, Boston, Massachusetts, United States of America; 7 Harvard-MIT Health Sciences and Technology, Cambridge, Massachusetts, United States of America; Charité, Campus Benjamin Franklin, Germany

## Abstract

**Background:**

CD4^+^ T-lymphocyte count (CD4 count) is a standard method used to monitor HIV-infected patients during anti-retroviral therapy (ART). The World Health Organization (WHO) has pointed out or recommended that a handheld, point-of-care, reliable, and affordable CD4 count platform is urgently needed in resource-scarce settings.

**Methods:**

HIV-infected patient blood samples were tested at the point-of-care using a portable and label-free microchip CD4 count platform that we have developed. A total of 130 HIV-infected patient samples were collected that included 16 de-identified left over blood samples from Brigham and Women's Hospital (BWH), and 114 left over samples from Muhimbili University of Health and Allied Sciences (MUHAS) enrolled in the HIV and AIDS care and treatment centers in the City of Dar es Salaam, Tanzania. The two data groups from BWH and MUHAS were analyzed and compared to the commonly accepted CD4 count reference method (FACSCalibur system).

**Results:**

The portable, battery operated and microscope-free microchip platform developed in our laboratory (BWH) showed significant correlation in CD4 counts compared with FACSCalibur system both at BWH (r = 0.94, p<0.01) and MUHAS (r = 0.49, p<0.01), which was supported by the Bland-Altman methods comparison analysis. The device rapidly produced CD4 count within 10 minutes using an in-house developed automated cell counting program.

**Conclusions:**

We obtained CD4 counts of HIV-infected patients using a portable platform which is an inexpensive (<$1 material cost) and disposable microchip that uses whole blood sample (<10 µl) without any pre-processing. The system operates without the need for antibody-based fluorescent labeling and expensive fluorescent illumination and microscope setup. This portable CD4 count platform displays agreement with the FACSCalibur results and has the potential to expand access to HIV and AIDS monitoring using fingerprick volume of whole blood and helping people who suffer from HIV and AIDS in resource-limited settings.

## Introduction

More than 30 million human immunodeficiency virus (HIV)-infected people live in the sub Saharan Africa, yet it is estimated that only one in ten persons infected with HIV has been tested and knows their HIV status [1], [2], [3], [4], [5], [6], [7]. Effective antiretroviral therapy (ART) for HIV has been available in developed countries for more than a decade and free of charge through philanthropic resources such as Bill and Melinda Gates Foundation, Clinton and Davis Duke, and governmental resources such as President's Emergency Fund for AIDS Relief (PEPFAR) [8]. However, in Africa, less than 4 out of 10 people who need a treatment are actually receiving ART [9]. Part of the problem associated with existing ART delivery services are the limitations of conventional methods to diagnose and monitor HIV-infected individuals living in rural communities.

According to the National AIDS control program (NACP) guidelines, ART is initiated for HIV-infected individuals with CD4^+^ T lymphocyte counts below 350 cells per microliter of blood [1], [2], [3], [6], [10], [11], and 500 cells per microliter threshold is commonly used to increase patient monitoring intensity [12]. Guidelines also require that patients should be regularly monitored for CD4^+^ T-lymphocyte counts at 6-month intervals. The current gold standard methodology for measuring CD4^+^ T-lymphocyte counts in whole blood is the fluorescent activated cell count and sorting systems (FACS) [3], [10], [13]. However, these systems face significant challenges in terms of scalability and applicability including high equipment cost, requirement for laboratory space, inadequate number of trained laboratory personnel, lack of reliable and regular preventive maintenance service and limited portability.

HIV infection has reached epidemic proportions in Tanzania with an estimated 1.3 million patients living with HIV/AIDS. Effective antiretroviral therapy (ART) for HIV has been available in Tanzania for more than a decade. However, it is estimated that less than 20% of all the infected individuals in Tanzania are currently receiving treatment, the most affected persons are living in rural and hard to reach communities. A microchip test that is portable and affordable has potential to impact HIV monitoring at all levels possibly with a higher impact at the dispensary level.

To increase access to HIV and AIDS care and to improve treatment outcomes requires development of inexpensive monitoring tools for resource-limited countries [13], [14], [15], [16]. The World Health Organization has stated that there is an urgent need for a handheld, point-of-care (POC), reliable, affordable CD4 T-cell count device for use in resource-limited regions [13]. While lower test costs (∼10 USD) are available in resource-limited countries at central laboratories, these costs still remain unaffordable for many patients. In addition, maintenance related expenses are also significant. Therefore, there is a need for affordable tests that can be performed at the POC in resource-scarce settings at all levels especially as we strive to move further down the healthcare delivery services.

The challenges with delivering healthcare at the POC in developed world settings include ease-to-use, sample processing, need for skilled health care workers, portability, and test turnaround time (**Table 1**) [17]. These factors have long constituted a bottleneck for recent microfluidic and lab-microchip type platforms to be translated to the bed-side as applicable diagnostic methodologies. These POC challenges adopt an insurmountable silhouette at the resource-constrained settings, where additional challenges are encountered. The difficulties associated with resource-limited POC healthcare delivery involve undependable electricity, demanding portability requirements on devices and readers, and limitations on the use of peripheral devices [3], [13], [17]. These interdependent challenges alter the parameters for POC device design. Therefore, we need to design new methodologies with practical thinking strategies to address problems that go beyond the developed world geared engineering and healthcare system [18]. These factors all broadly define the poor performance encountered by well-operating, lab-designed machinery in such resource-limited settings. Furthermore, fluorescent labeling of cells for detection and enumeration present significant challenges in these settings [6], [11], [17], [19]. Therefore, label-free approaches with simple imaging methods are needed at the resource limited POC settings. Taking on these intriguing biomedical, engineering and design challenges [20], [21], we here report clinical results from Tanzania using a POC CD4 count microchip, which is an inexpensive (<$1 material costs), disposable device that uses a volume of whole blood sample (<10 µl) without any sample pre-processing. Our test results in BWH and Tanzania have shown that the device produces CD4 T-cell counts that are statistically comparable to FACSCalibur results.

**Table 1:** Comparison for the design requirements and operational demands of a POC monitoring device based on World Health Organization (WHO) Standards for POC healthcare delivery at resource-scarce settings, and conventional devices at developed world settings.

	Resource-Scarce Settings	Developed World Settings
**Cost**	Inexpensive per test; disposable; maintenance free or minimal inexpensive maintenance	Expensive per test; costly to maintain
**Personnel Needs**	Minimally training; easy to maintain and operate	Requires highly-trained personnel
**Environmental Conditions**	Functional at high temperatures and high humidity conditions	Room temperature and regular humidity levels
**Infrastructure**	Battery operable without dependence on constant electrical supply	Needs advanced and reliable infrastructure without power outages
**Accessibility**	Portable; deliverable to end users as a handheld system without a need for centralized hospitals or clinics	Performed at established hospitals and clinics beyond the district level
**Accuracy & Precision**	Moderate to high to satisfy the minimal requirements of clinical decision making	High
**Throughput**	High	High

## Methods

### Study population and sample collection

De-identified left-over EDTA whole blood samples were obtained from HIV-infected female and male patients between the ages of 14 and 37, who were enrolled in the Harvard PEPFAR supported HIV and AIDS care and treatment centers in Dar es Salaam, Tanzania. To minimize additional time and workload of both the patient and clinic staff our study team used whole blood samples collected from de-identified HIV-infected patients during routine patient care visits at the site clinics. Left over whole blood samples for testing were taken from existing routine collection procedures during processing at the central laboratory at Muhimbili University of Health and Allied Sciences (MUHAS), in Dar es Salaam, Tanzania, which did not require an additional blood draw from patients. Out of 130 samples included in the testing, 114 de-identified samples were selected randomly by the study coordinator at the central laboratory, among the samples that were received from each of the site clinics on a daily basis for consecutive two weeks. Sixteen de-identified left over blood samples were obtained from Brigham and Women's Hospital in Boston, USA. The existing HIV and AIDS care and treatment program specimen tracking system was used to verify dispatch and receipt of the whole blood specimens.

### Ethics

The studies described here were performed under the approval of the following Institutional Review Boards (IRB): Harvard School of Public Health, Partners Human Research Committee, and Muhimbili University of Health and Allied Sciences Directorate of Research and Publications. Prior to the studies, the IRBs approved ethical clearance and determined that the research activities did not meet the definition of human subjects research for the following reasons: 1) there was no intervention of interaction with a living person for this research, and 2) there was no identifiable personal information obtained for this research in a form that can be associable with the individuals from whom the blood samples were obtained. This determination was based upon the U.S. Department of Health & Human Services Office for Human Research Protections Human Subject Regulations Decision Charts.

### Microfluidic CD4 T-cell Count System

**Figure 1:**
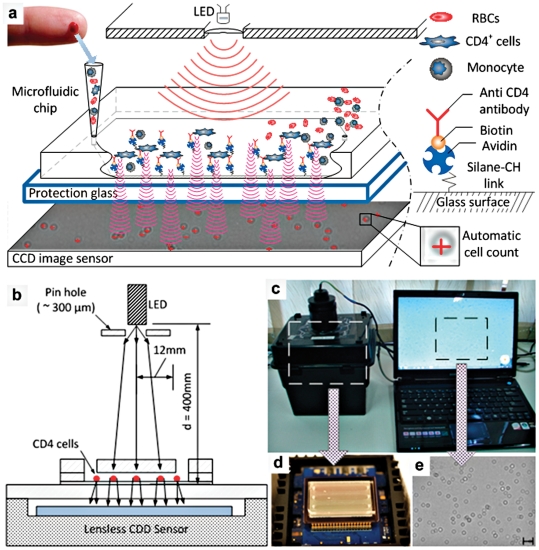
Depiction of the working principle of CD4^**+**^ T-lymphocyte counting microchip platform for the POC. (**a**) Shadow images of captured cells were obtained using a large area CCD image sensor (24×34 mm, 10 mega pixel). A pinhole LED (Light Emitting Diode) was used as a light source. The POC platform has following two main processes. First, a microfluidic chip captured target CD4^+^ cells from unprocessed fingerprick volume of HIV-infected whole blood by anti-CD4 antibody which was immobilized on the microchip surface. Second, the captured cells were imaged using the wide field of view (FOV) lensless CCD platform within a second. White light generated from LED light source went through a 100 µm pinhole and illuminated captured cells. Cell shadows were automatically detected and rapidly counted by automated image recognition software on a portable laptop computer. (**b**) Schematic representation and drawing of the lensless imaging system for microfluidic CD4 count, (**c**) Photograph of entire POCT CD4 imaging system, (d) microfluidic CD4 chip on top of CCD sensor inside a black box, (e) lensless imaging and magnified image represents the diffracted shadow signal of the cell shape. Scale bar is 200 µm. Entire platform setup and operational details were shown in **Text S1**.

The microchip that captured CD4 cells was directly imaged with a portable, battery operated CCD imaging platform and cells were counted by automatic cell count software within a minute without the need for a labeling process (**Fig. 1**). To image shadow patterns of captured cells (**Fig. 1a**) with the CCD image sensor (**Fig. 1b**, KODAK, KAI-11002, Rochester, NY), one gray color image of the entire channel surface was taken (**Fig. 1c–e**). The sensor featured more than 11 million square pixels (9 µm size), across the active sensor array area, 37.25 mm×25.70 mm (**Fig. 1d**). The large active sensor area of the CCD enabled us to use standard microscope cover slides (24 mm×35 mm×0.10 mm) with a thickness of 0.10∼0.15 mm as the microfluidic channel surface. The white light, emitted by a LED (276-0024, high brightness white LED, RadioShack), passes through the PMMA cover, reaches bottom glass surface of the microfluidic channel and the captured cells on this surface. Lensless image represented the diffracted shadow signal of the cell shape (**Fig. 1e**). When the distance between the captured cells and CCD surface was increased, the ring diameter of shadow image increased. This effect was observed till the signal-to-noise between the shadow and background light intensity reached to the CCD detection limit. The system was designed to be adopted for POC testing, which was composed of a black box containing a CCD sensor and a mobile computer that weighed about 1 kg.

### Fabrication and Operation of the Portable Microchip Platform

**Figure 2:**
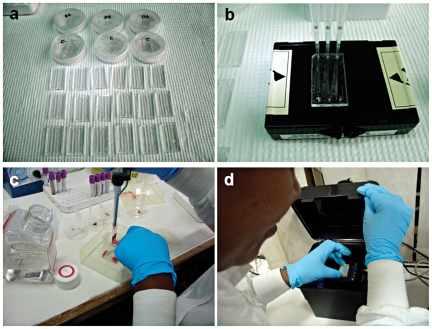
Overview of Microfluidic CD4 counting chip preparation, blood injection, and lensless imaging procedures by minimally trained personnel at MUHAS. (**a**) Unpacking of the microfluidic chips, (**b**) antibody injection, (**c**) one step blood injection, (**d**) microfluidic CD4 count chip imaging using a point-of-care portable, battery operated lensless CCD based imaging platform.

Detailed description and illustration of device fabrication (**Table S1** and **Fig. S1**), preparation (**Fig. S3** and **Table S2**), and the standard operating procedures (**Table S3**) can be found in **Text S1**, which were based on our previous studies [14]. The microfluidic whole blood CD4 counting operation is outlined in **Figure 2**, which was performed by minimally trained personnel at MUHAS (**Table S3**). Briefly, the microfluidic chips were removed from their vacuum sealed packages (**Fig. S2** and **Fig. 2a**), the capture antibody was injected into the chips followed by a brief washing step (**Table S2** and **Fig. 2b**), a one step whole blood injection was performed (**Fig. 2c**), and the microfluidic chip was imaged using a portable CCD based imaging platform (**Fig. 2d**). The gravitational flow required filling 50 µL of whole blood into the pipette directly on top of the inlet of the device without additional sample handling or processing (**Fig. 2b&c**). The pipette was removed from the inlet as soon as the channel got fully filled with the whole blood indicated by the red color. This process led to an estimated 8 µL of whole blood to pass through the device in MUHAS tests. In BWH tests, the volume of blood introduced into the channels was controlled with automated pipettes to be 6 µL per channel. The cell counts obtained from the microchips were normalized with the total blood volume to convert the results to number of cells per microliter of whole blood.

### Automated CD4 Cell Counting in CCD images

**Figure 3:**
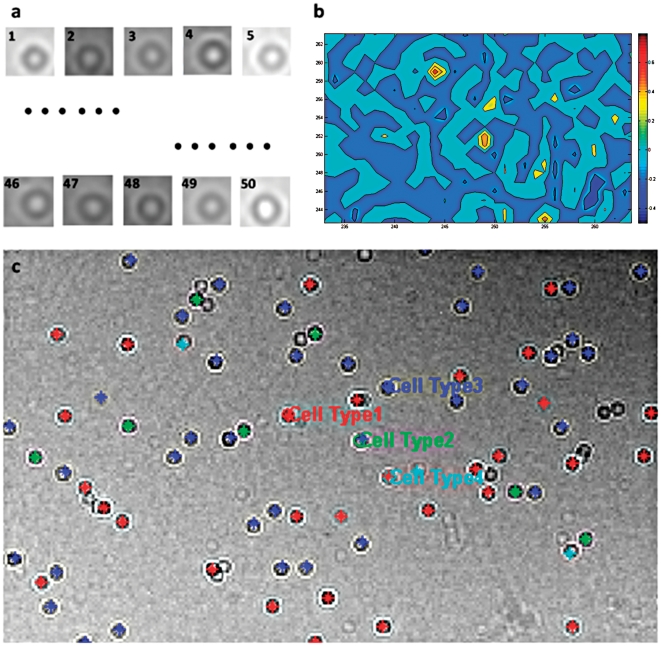
The typical output of automatic CD4 cell counting program based on pattern matching. (**a**) library images of cell types (a total 50 different images), (**b**) matching values compare an obtained shadow image and cell library images, (**c**) result of cell shadow image recognition using four different types of library cell patterns. The detected cells were marked with color coding to each library image that they were best matched using standard pattern recognition matching methods in MATLAB.

An automated cell counting program was developed in MATLAB (Mathworks Inc., Natick, Massachusetts) to resolve a threshold signal level, which then determined the boundaries between the cell membranes and the background. The CCD shadow images were used to count the cells and to characterize a distribution of captured cells using image recognition based automated counting software. Automatic cell counting program was designed based on pattern matching (**Fig. 3a**). In this method, randomly selected CCD images of cells (a library of 50 images) were evaluated to match the specific cell type. The matching values from shadow imaging were obtained and quantified (**Fig. 3b**). Based on a 50% matching value threshold between a library image and the shadow microchip image, the captured cells were detected and counted (**Fig. 3c**). Total number of CD4+ T-lymphocytes obtained from the microfluidic device by counting cell shadow images and the flow cytometry results (as a gold standard) were compared.

### Determination of CD4 Counts In Whole Blood Samples with FACS

The whole blood sample was analyzed using similar reference flow cytometry method at the two study sites to enumerate the CD4+ T-lymphocytes. The flow cytometric measurements were performed on a FACSCalibur (Beckton Dickinson Immunocytometry System (BDIS, San Jose, CA) instrument using BD CellQuest Pro Software. The flow cytometer was calibrated using standardized bead kit (FACS TruCOUNT™, BD Biosciences, Mountain View, CA). CD4-Chex Plus BC (Streck, NE, USA) controls (low and high) were run every day in the FACSCalibur system at MUHAS lab before patient samples are run to ensure accuracy and reliability of lab results. MUHAS laboratory also participates in the proficiency testing program called UK NEQAS (United Kingdom National External Quality Assessment Service) where a panel of whole blood samples were received, analyzed in the FACSCalibur system with a good performance report. The CD4 counts were reported as cells per microliter of whole blood sample.

### Statistical Analyses

We tested de-identified left over whole blood samples collected from adult patients at BWH (n = 16) and at MUHAS (n = 114). We validated CD4^+^ T cell counts from the microchips in comparison to the FACSCalibur results using the Pearson product-moment correlation coefficient and Bland-Altman analysis. Statistical significance threshold was set at 0.01 (p<0.01). The analyses were performed with Minitab (Release 14, Minitab Inc., State College, PA, USA). By validating a microchip CD4 count platform against the FACSCalibur system, we aimed to analyze how much the microchip method is likely to differ from the reference method. The used validation procedures were as follows [14]: 1) Assessing the linear regression between the microchip and FACSCalibur counts, 2) assessing the Pearson product moment correlation coefficient, r, between the two methodologies, 3) Computing the difference and the mean between FACSCalibur and microchip device for each specimen, 4) Generating a scatter plot with differences for each specimen on the Y-axis plotted against the means for each corresponding specimen on the X-axis, 5) Calculating the average difference between microchip devices against the FACSCalibur count and the standard deviation of the differences. The regression curves were generated using results obtained from microchips each treated with the similar volume of blood sample. The microchip measurements were normalized by the estimated blood volume processed inside microfluidic channel (8 µL) based on the gravitational flow. The agreement between the two methods was evaluated by Pearson product-moment correlation coefficient and Bland-Altman analysis with statistical significance threshold set at 0.01 (p<0.01). The Bland-Altman comparison analysis method was used for the repeatability of the method using residual analysis by comparison to the gold standard method. The coefficient of repeatability was calculated as 1.96 times the standard deviations of the differences between the two measurements. In this analysis, a mean difference of zero infers that the tested approach is unbiased with respect to the standard. A clinically acceptable range would indicate the range within which the difference would fall approximately 95% of the time. If the mean difference and the limit of agreement are within the clinically acceptable range, then the tested technique is deemed comparable to the standard technique.

## Results

**Figure 4:**
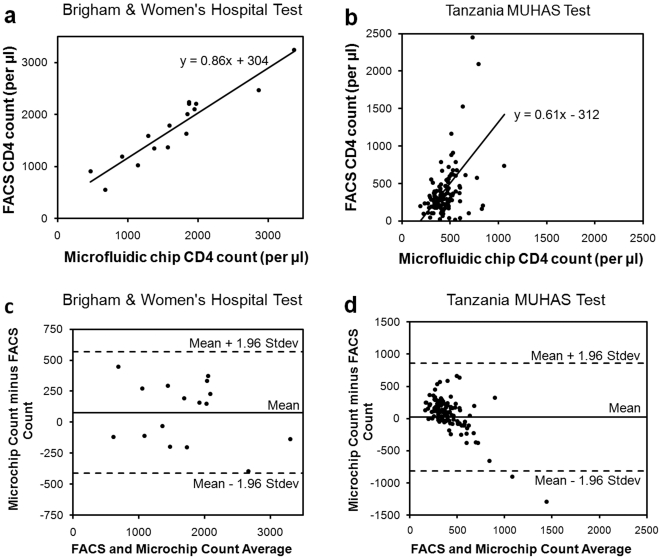
Comparison of microchip and Fluorescent Activated Cell Sorting (FACS) CD4^**+**^ T-lymphocyte counts for HIV-infected patient whole blood samples. The measurements were performed at BWH (Brigham and Women's Hospital, Boston, MA) and MUHAS (Muhimbili University of Health and Allied Sciences, Dar es Salaam, Tanzania). FACSCalibur counts were used as the gold standard to compare and validate the microchip counts. (**a**) Microchip measurements highly correlated with FACSCalibur measurements when conducted at an established hospital setting at BWH (y = 0.86x+304, correlation coefficient: 0.94, p<0.01). (**b**) When the microchips were shipped to Tanzania and the measurements were performed at MUHAS, a correlation between the microchip CD4 counts and the FACS counts were observed (y = 0.61x+312, correlation coefficient: 0.49, p<0.01). (**c**) Bland-Altman Analysis between the microchip and FACS counts did not display an evidence for a systematic bias for BWH measurements. The mean bias was +76 cells per µL of blood in microchip counts compared to FACS counts at BWH. (**d**) Bland-Altman Analysis showed a bias towards higher measurements in MUHAS results, while the mean bias was as low as +23 cells per µL of blood in microchip counts compared to FACS counts.

An overview of microfluidic CD4 T cell count device and whole blood processing procedure is shown in **Fig. 1**. Shadow images (**Fig. 1a**) of captured cells from unprocessed volume of whole blood were obtained using a large area CCD image sensor (**Fig. 1b–e**) within a second by minimally trained personnel at Tanzania (**Fig. 2**). Cell shadows were automatically detected and counted by image recognition software (**Fig. 3**). Microchip measurements correlated significantly with FACSCalibur counts, when they were conducted at BWH (**Fig. 4a**, r: 0.94, p<0.01). When the microchips were transported to Tanzania over 48 hours on dry ice in a plane setting, the correlation coefficient between the microchip and the FACSCalibur system counts were statistically significant, however, with a lower correlation coefficient for the measurements that were performed at MUHAS laboratory (**Fig. 4b**, correlation coefficient: 0.49, p<0.01). The result of the Bland-Altman Analysis showed that no dependency of the captured CD4 T cell count on the measurement magnitude was observed in the case of BWH measurements (**Fig. 4c**). The mean bias was +76 cells/µL of blood in microchip counts compared to FACS counts at BWH. On the other hand, Bland-Altman Analysis showed a bias towards higher measurements in MUHAS results, while the mean bias was as low as +23 cells/µL of blood in microchip counts compared to FACS counts (**Fig. 4d**).

## Discussion

In the developed CD4 T cell count system, even though the resource-limited environmental factors were observed to adversely affect the operation, microchip CD4 T cell counts showed a correlation and agreement with the FACSCalibur results. The microchip CD4 counts were observed to highly correlate with the FACSCalibur counts (correlation coefficient: 0.94, p<0.01) when the measurements were performed at BWH by highly trained healthcare personnel. On the other hand, microchip CD4 counts that were performed at MUHAS using blood samples showed a significant but lower level of correlation with the FACSCalibur results (correlation coefficient: 0.49, p<0.01). These observed differences in correlation between the BWH and MUHAS tests could be explained by the environmental and the operator-dependent factors present under the real world conditions of resource limited settings.

The tests performed at BWH were carried out by highly trained individuals on microfluidic techniques. The operators in Tanzania were trained over a short course lasting about 30 minutes. These operators followed clearly written standard operating procedures (**Text S1**). For instance, automated pipettes were used at BWH tests, which enabled accurate and uniform flow control through the microfluidic channels at every stage of operation. It has been shown that uniform flow yields improved results compared to manual or gravity driven flow in microfluidic systems as also reflected in our results. In addition, the microchips that were used at BWH were freshly built and did not undergo the travel conditions and long durations of intercontinental shipment (24–72 hours). The microchips that were used at MUHAS, which were built at BWH and transferred to Tanzania on dry ice, could potentially influence the CD4 capture and count efficiency. All these factors reflected the challenges of delivering affordable healthcare in resource limited settings. Therefore, we converge that these factors need to be further evaluated and resolved to increase the operational efficiency of POC devices.

It should be noted that the BWH patients were already under ART treatment and therefore had high CD4 counts (between 542 and 3236 CD4 counts per microliter of blood, **Fig. 4a&c**). The Tanzania patient samples showed lower range of CD4 counts (**Fig. 4b&d**). Therefore, we have evaluated the sensitivity of the developed microfluidic CD4 count system with the Tanzania patient samples for the clinically relevant CD4 count threshold values (i.e., <350 cells/µl to initiate ART, and <500 cells/µl to increase patient monitoring intensity) [12]. The CD4 count microfluidic chip developed in this study displayed 41% sensitivity in detecting the CD4 counts less than 350 cells/µl in Tanzania blood samples after correcting for the bias (+23 cells/µl) determined with Bland-Altman analysis (**Fig. 4d**). On the other hand, the detection sensitivity was as high as 87% for CD4 counts less than 500 cells/µl after corrections for the bias. The lower sensitivity in the case of 350 cells/µl threshold was mostly due to the storage conditions of the microchips, where we preferred not to transfer them on cold chain to see the real impact under non-ideal storage conditions. Hence, antibody denaturation most likely caused lower capture efficiency and detection sensitivity than the BWH results as we had anticipated. As a solution to this problem, it has been shown that sensitive immunologic and biochemical reagents in microfluidic systems (e.g., antibodies) can be lyophilized to significantly increase the shelf life of the test system [22], [23].

The microchip platform presented here weighed 1 kg, and it was attached to a laptop system to acquire the shadow images of captured cells and count the CD4 T cells using automated software without the need for cell labelling. This whole system was battery operable and portable. The CD4 count system has the potential to be further miniaturized by replacing the laptop and the imaging setup with smaller handheld units such as cell phones with cameras indicating the future potential of such a system. Further, the microchip can be integrated with a simple aspiration system, where a single drop of blood can be introduced into the microchip. These developmental stages can bring this portable system closer to a commercially available product that can be broadly used in resource-limited settings. Further, we anticipate that these cost reductions in medical testing and enhancements in POC diagnostics and monitoring technologies will also impact the developed world healthcare delivery posing a broad interest for the general public.

In conclusion, we have performed for the first time label-free (fluorescent-free) CD4 T-lymphocyte counts from HIV-infected patient blood with a disposable microchip system in the USA and in Tanzania. Our results showed that portable CD4 capture and counting devices are feasible and applicable at the POC settings. The capture efficiency of the developed microchips was observed to be dependent on cell concentration, environmental factors and operational variations. Therefore, detection systems specifically designed for POC must be tested under the full conditions of resource limited settings for reliable evaluation and assessment.

## Supporting Information

Figure S1 **Fabrication method for the microfluidic chips.** Components (PMMA and double sided adhesive) were machined by using a 30W CO_2_ laser cutter and bonded on a glass slide.
(TIF)

Figure S2 **Microfluidic CD4 chip packaging for intercontinental logistics.** (**a**) The inlet and outlet ports of the microfluidic chips were sealed with adhesive tapes and vacuum sealed in packaging. (**b**) The microfluidic chips were further placed in parafilm sealed Petri dishes to further enhance the transportation safety.
(TIF)

Figure S3 **A brief description of the surface chemistry protocol to immobilize antibodies in microfluidic chips.** The surface modification method was based on silane bonding with an activated glass surface (i.e., 3MPS, GMBS, Neutravidin, and anti-CD4 antibody). Scale bar is 4 mm.
(TIF)

Table S1 **Standard Operation Procedure (SOP) for fabricating the microfluidic chips prior to applying the surface chemistry.**
(DOC)

Table S2 **Standard Operation Procedure (SOP) for surface chemistry in microfluidic chips at the point of care prior to blood testing.**
(DOC)

Table S3 **Standard Operation Procedure (SOP) for blood testing at the point of care with CD4 counting microfluidic chips.**
(DOC)

Text S1 (DOC)
