# Supplementary figures and images for: Enumeration of CD4+ T-Cells Using a Portable Microchip Count Platform in Tanzanian HIV-Infected Patients
Source: PLoS One. 2011 Jul 6;6(7):e21409. doi: 10.1371/journal.pone.0021409 (PMC3130745; doi:10.1371/journal.pone.0021409)

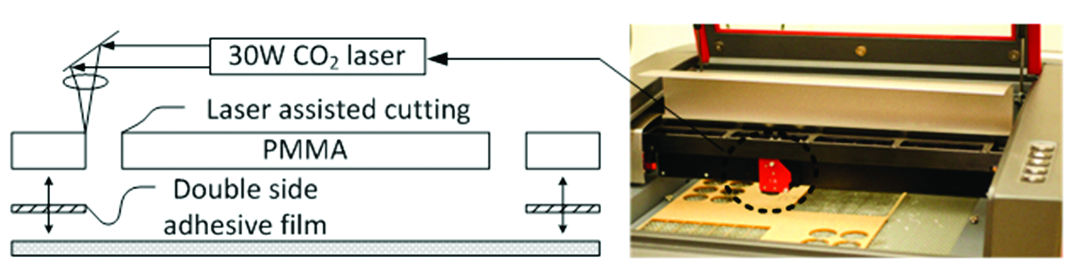

Supplement: Figure S1 — Fabrication method for the microfluidic chips. Components (PMMA and double sided adhesive) were machined by using a 30W CO2 laser cutter and bonded on a glass slide. (TIF) [file pone.0021409.s001.tif]

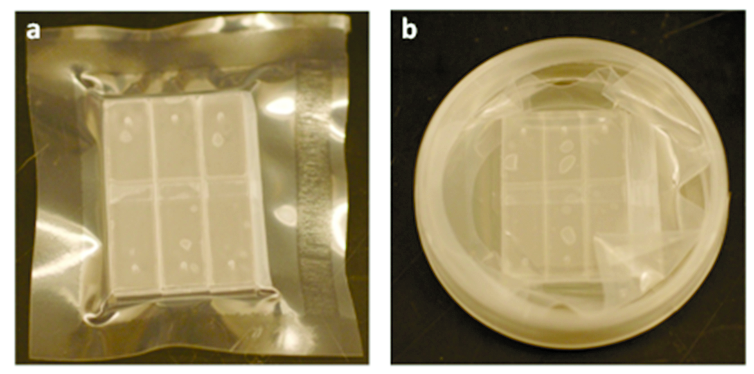

Supplement: Figure S2 — Microfluidic CD4 chip packaging for intercontinental logistics. (a) The inlet and outlet ports of the microfluidic chips were sealed with adhesive tapes and vacuum sealed in packaging. (b) The microfluidic chips were further placed in parafilm sealed Petri dishes to further enhance the transportation safety. (TIF) [file pone.0021409.s002.tif]

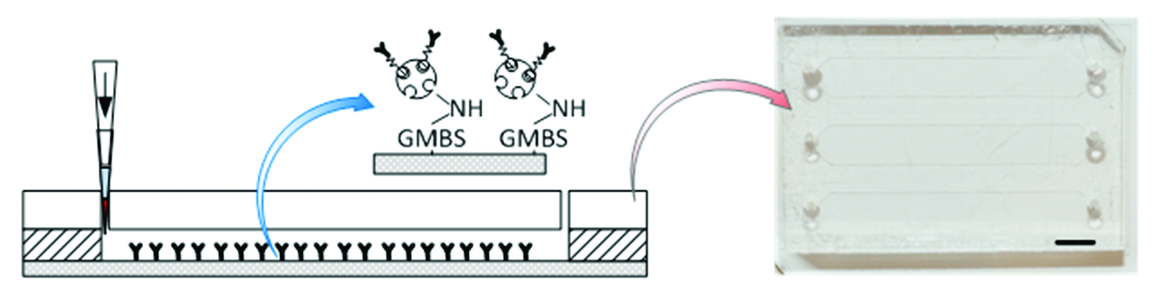

Supplement: Figure S3 — A brief description of the surface chemistry protocol to immobilize antibodies in microfluidic chips. The surface modification method was based on silane bonding with an activated glass surface (i.e., 3MPS, GMBS, Neutravidin, and anti-CD4 antibody). Scale bar is 4 mm. (TIF) [file pone.0021409.s003.tif]
