# Supplementary material for: Enumeration of CD4+ T-Cells Using a Portable Microchip Count Platform in Tanzanian HIV-Infected Patients
Source: PLoS One. 2011 Jul 6;6(7):e21409. doi: 10.1371/journal.pone.0021409 (PMC3130745; doi:10.1371/journal.pone.0021409)
Supplement: Table S1 — Standard Operation Procedure (SOP) for fabricating the microfluidic chips prior to applying the surface chemistry. (DOC) [file pone.0021409.s004.doc]

**Table S1**.

| ***Step*** | ***Step Description*** | ***Methodology*** | ***Conditions*** | ***Solutions/specification*** |
| --- | --- | --- | --- | --- |
| **0** | Cleaning glass substrate | Ultrasonic in IPA | 30min | 1. Dry with nitrogen gas  2. Keep in petridish |
| **1** | Plasma treatment | Glass, Air Plasma | RT | 1mm gap between glass and electrode, 1 mm/sec, 2 sweeps |
| **2** | Assembly | PMMA Channels to Glass | RT | - |
| **3** | Silanization | Injection 15 ul per channel of silanization solution | Incubate 30 minutes @ RT | 1. Silanization solution  2. Keep devices in petridish to prevent evaporation of silanization solutions |
| **4** | Rinse I | Rinse with 30ul Ethanol | RT | EtOH |
| **5** | GMBS | Injection 15 ul per channel of GMBS solution | Incubate 15 minutes @ RT | 1. GMBS solution  2. Seal with Parafilm to prevent evaporation of silanization solutions |
| **6** | Rinse I | Rinse with 30ul Ethanol | RT | EtOH |
| **7** | Rinse II | Change 🡪 Rinse with 30ul PBS | RT | 1. Make sure that white residue will not be shown  2. EtOH to PBS phase change |
| **8** | Neutravidin | Injection 15ul per channel of Neutravidin solution | O.N. or 1hour @ 4^o^C | Neutravidin solution |
| **9** | Rinse II | Rinse with 30ul PBS | RT | PBS |
| **10** | BSA passivation | Injection 15ul per channel of BSA solution | Incubate 10 minutes @ RT | BSA : PBS= 1:100 |
| **11** | Rinse II | Rinse with 30ul PBS | RT | PBS |

RT: Room Temperature

PBS: Phosphate Buffered Saline Solution

EtOH: Ethyl Alcohol
