# Supplementary material for: Enumeration of CD4+ T-Cells Using a Portable Microchip Count Platform in Tanzanian HIV-Infected Patients
Source: PLoS One. 2011 Jul 6;6(7):e21409. doi: 10.1371/journal.pone.0021409 (PMC3130745; doi:10.1371/journal.pone.0021409)
Supplement: Table S2 — Standard Operation Procedure (SOP) for surface chemistry in microfluidic chips at the point of care prior to blood testing. (DOC) [file pone.0021409.s005.doc]

**Table S2**.

| ***Step*** | ***Step Description*** | ***Methodology*** | ***Conditions*** | ***Solutions/specification*** | ***Accumulated***  ***Process time*** |
| --- | --- | --- | --- | --- | --- |
| **1** | Start | Should be started 2 hours before getting blood sample for parallel processing | | | |
| **2** | Wash | Fill with 100ul/channel PBS | Wait minimum 10 min | Inject and release pipette tip | 10 min |
| **3** | Antibody | Injection 50ul/channel of anti-CD4 solution | 1hour at room temperature | anti-CD4 solution | 1 hour 10 min |
| **4** | Rinse | Fill with 100ul/channel PBS | 10 min at room temperature | Dilute with PBS 1:10 | 1 hour 20 min |
| **5** | BSA passivation | Fill with 100ul/channel PBS | 1hour at room temperature | BSA : PBS = 1% BSA | 2 hours 20 min |
