# Supplementary material for: Enumeration of CD4+ T-Cells Using a Portable Microchip Count Platform in Tanzanian HIV-Infected Patients
Source: PLoS One. 2011 Jul 6;6(7):e21409. doi: 10.1371/journal.pone.0021409 (PMC3130745; doi:10.1371/journal.pone.0021409)
Supplement: Table S3 — Standard Operation Procedure (SOP) for blood testing at the point of care with CD4 counting microfluidic chips. (DOC) [file pone.0021409.s006.doc]

**Table S3**.

| ***Step*** | ***Step Description*** | ***Methodology*** | ***Conditions*** | ***Solutions/specification*** | ***Accumulated***  ***Processing time*** |
| --- | --- | --- | --- | --- | --- |
| **1** | Blood injection | Injection 50ul/channel | 5 ul/min | 1. Check blood front reach to outlet port  2. Remove pipette tip after the blood front reaching the outlet port | 2 min |
| **2** | Mechanical filter | Injection 100 ul/min with cell fixing solution | 20 ul/min | Cell fixing solution, 3 time wash | 5 min |
| **3** | Clean surface | Wipe the microchip surface with ethanol tissue before imaging | - | Inspect whether there is some artifact or not | 8 min |
| **4** | CCD imaging | Place the microchip in the CCD imaging setup and acquire the image | - | 1. Clean CCD surface with ethanol  2. 420 ms exposure time | 9 min |
| **5** | Automated Counting | Run the shadow images through the software code | - | 10-20 second processing time | 10 minutes |
