# Supplementary material for: Enumeration of CD4+ T-Cells Using a Portable Microchip Count Platform in Tanzanian HIV-Infected Patients
Source: PLoS One. 2011 Jul 6;6(7):e21409. doi: 10.1371/journal.pone.0021409 (PMC3130745; doi:10.1371/journal.pone.0021409)
Supplement: Text S1 — (DOC) [file pone.0021409.s007.doc]

Supplementary Information

Enumeration of CD4^+^ T-cells using a portable microchip count platform in Tanzanian HIV-infected patients

SangJun Moon^1^, Umut Atakan Gurkan^1^, Jeffrey Blander^1,2^, Wafaie W. Fawzi^3^, Said Aboud^4^, Ferdinand Mugusi^5^, Daniel R. Kuritzkes^6^, and Utkan Demirci^1,7,#^

^1^ Demirci Bio-Acoustic MEMS Laboratory, Center for Biomedical Engineering, Brigham and Women’s Hospital, Harvard Medical School, Boston, MA, USA.

^2^ Department of Epidemiology, Harvard Medical School, Boston, MA, USA

^3^ Department of Nutrition, Department of Epidemiology, Department of Global Health and Population, Harvard School of Publich Health, Boston, MA, USA

^4^ Department of Microbiology and Immunology, Muhimbili University of Health and Allied Sciences, Dar es Salaam, Tanzania

^5^ Department of Internal Medicine, Muhimbili University of Health and Allied Sciences, Dar es Salaam, Tanzania

^6^ Section of Retroviral Therapeutics, Brigham and Women's Hospital, Harvard Medical School, Boston, MA, USA.

^7^ Harvard-MIT Health Sciences and Technology, Cambridge, MA, USA.

# Corresponding Author, [udemirci@rics.bwh.harvard.edu](mailto:udemirci@rics.bwh.harvard.edu)

**MICROFLUIDIC CD4 COUNT CHIP FABRICATION AND PACKAGING FOR LONG-DISTANCE LOGISTICS**

**Fabrication of Microfluidic CD4 Count Chips**

A schematic of the fabrication process is shown in **Figure S1** and detailed in **Table S1**. For the microfluidic device, a 1/8" thick clear cast acrylic sheet (PMMA, Polymethymetacrylate) and a 50 µm thick optically clear double sided adhesive sheets (Model number, 8142) were obtained from McMaster Carr Supply Co. Inc (Los Angeles, CA) and 3M (St. Paul, MN), respectively. The PMMA and double sided adhesive film were cut by the X-660 Laser Platform (Universal Laser Systems Inc., Scottsdale, AZ) with a CO_2_ laser to form the inlet, outlet, and the fluidic channel. The laser machined PMMA and the adhesive film were aligned and assembled manually with a one step process. The PMMA with double sided adhesive was then mounted on a glass slide to form the microfluidic channels.

**Material Costs for Microchip Fabrication**

The microchips were manufactured with PMMA bonded to glass slides with a double sided adhesive to make the chip easy to fabricate reliably and repeatably in a standard laboratory setting. The repeatability and mass production of the device could be enabled by building the device using injection molded plastic or alike polymers allowing batch fabrication. The fluid volumes necessary for staining of a single microchip were at the order of microliters due to the small volume of the microfluidic (<10 µl). Therefore, the cost associated with biological compounds such as antibodies can potentially be decreased to less than 10 cents in a batch fabrication process. The microchip design presented here has the potential to offer an overall cost of less than 1 USD.

**Device Packaging for Long-Distance Logistics**

As shown in **Figure S2**, microfluidic CD4 chip was packaged for intercontinental logistics from Boston, USA to Dar Es Salam, Tanzania. Three methods were used to keep the device functional without dehydration. All inlet and outlet ports were sealed before packaging with a removable 3M tape (Scotch Magic Tape, Cat-810, 3M) to prevent liquid leaking from the channels during shipping process. Vacuum seal packaging (S-14569, ULINE) was used to seal devices as shown **Figure S2a**, which was processed using a conventional vacuum packaging instrument (H-1075 vacuum sealer, ULINE). Parafilm^TM^ (PM-999, parafilm) was used to seal the containers (1007 Falcon, Beckman Dickinson), which encased a chip and 1ml of excessive PBS (**Fig. S2b)**.

**SURFACE MODIFICATION OF MICROFLUIDIC CHANNELS AND BLOOD TESTING**

**Materials for Surface Modification and Solution Preparation**

Ethanol (200 proof) and glass slides (24×35 mm, no. 1) were obtained from Fisher Scientific (Fair Lawn, NJ). Dimethyl sulfoxide (DMSO), Lyophilized bovine serum albumin (BSA), and a glovebox for handling the moisture-sensitive silane were acquired from Aldrich Chemical Co. (Milwaukee, WI). Phosphate buffered saline (PBS) 1× was purchased from Gibco (Grand Island, NY). 3-Mercaptopropyl trimethoxysilane was obtained from Gelest (Morrisville, PA). The coupling agent GMBS (N-y-maleimidobutyryloxy succinimide ester) were obtained from Pierce Biotechnology (Rockford, IL). Biotinylated mouse anti-human anti-CD4 (clone 13b8.2) was obtained from Beckman Coulter (Somerset, NJ). Solutions of 3-mercaptopropyl trimethoxysilane in ethanol, silanization solution, and a 1 µmol/ml of GMBS stock solution (50mg of GMBS is dissolved in 0.5mL of DMSO) in ethanol, GMBS solution, were prepared under a nitrogen atmosphere inside a glove box. A 10 mg neutravidin in 1 ml of PBS (neutravidin solution) was reconstituted as recommended by the manufacturer. 10% (v/v) Biotinylated mouse anti-human anti-CD4 solution in PBS containing 1% (w/v) BSA, CD4 antibody solution and 10% (v/v) solutions of BD FACS lysing Solution , cell fixing solution, were prepared.

**Surface Preparation and Modification of the Microfluidic Channels**

A schematic of the surface chemistry process is shown in **Figure S3**. After antibody (anti-CD4 antibody) being immobilized, microchip was packaged following as described in **Table S1 & S2** (standard procedure for surface modification). First, glass slides were treated with an oxygen plasma (100 mW, 1% oxygen, 60 s) in a PX-250 plasma chamber (March Instruments, Concord,MA) and then rapidly assembled with PMMA to form a microchannel. The microchannels were treated with silanization solution for 30 min at room temperature, followed by incubation with GMBS solution for 15 minutes at room temperature. Next, neutravidin was immobilized to GMBS by incubating the chamber surfaces with neutravidin solution for at least 1 hour PBS inside a refrigerator (4°C) or stored overnight in a refrigerator. The packaged chips were injected with CD4 antibody solution (**Table S2)**, and was allowed to react for 30 minutes at 4°C. One more CD4 injection was performed to increase capture efficiency. After each surface modification step, the channel surface was rinsed with PBS. Entire process to immobilize anti-CD4 antibody takes 2 hours and 20 minutes per chip.

**CD4+ T-lymphocyte Capture in Microfluidic Channels**

After preparing microfluidic CD4 chips, blood sample was introduced by a gravity driven method into the channels at MUHAS (**Table S3)**. Gravitational flow approach required filling 50 µL of whole blood into a pipette directly positioned on the inlet of the microchip without additional sample handling or processing. The pipette was removed from the inlet when the microchannel got filled with the whole blood, which was indicated by the full development of red color inside the channel. This step resulted in an estimated 8 µL of whole blood to be processed through the device per channel. Further, gravitational flow was used to introduce PBS solution into the microchannels to wash out the uncaptured cells. This pipetting and gravity based simple flow approach was conveniently utilized at MUHAS by minimally trained personnel. However, at BWH automated volume controlled pipette systems were used to introduce whole blood samples into the microfluidic channels by trained personnel. The automated pipetting resulted in processing of 6 µL whole blood in the channels. The volume of processed whole blood was chosen such that cell adhesion on the surfaces reached a pseudo-steady state. The cells were then fixed on the surfaces by incubating with cell fixing solution for 3 minutes and rinsed with PBS. Next, the microfluidic chips were transferred into the portable CCD system for imaging and enumeration of the captured cells.
